# Supplementary material for: Heterogeneities in infection outcomes across species: sex and tissue differences in virus susceptibility
Source: Peer Community J. Author manuscript; Available in PMC 2023 Feb 20. (PMC7614206; doi:10.24072/pcjournal.242)
Supplement: Supplementary Material [file EMS164615-supplement-Supplementary_Material.pdf]

Supplementary information

Table S1: Full list of species used in the sex difference experiment and their rearing food for stock populations. All cornmeal and proprionic medium have dried yeast sprinkled onto the surface of the food, other food types do not unless stated below. The recipes for the food types are described here https://doi.org/10.6084/m9.figshare.21590724.v1

Species	Food
D.affinis	Malt
D.americana	Malt
D.ananassae	Cornmeal
D.arizonae	Banana
D.buzzatii	Malt
D.erecta	Malt + yeast
D.flavomontana	Malt + yeast
D.hydei	Cornmeal
D.immigrans	Malt + yeast
D.lacicola	Malt
D.littoralis	Banana
D.mauritiana	Proprionic
D.melanogaster	Cornmeal
D.montana	Malt + yeast
D.novamexicana	Banana
D.obscura	Proprionic
D.persimilis	Malt
D.prosaltans	Proprionic

D.pseudoobscura	Malt
D.putrida	Proprionic
D.santomea	Cornmeal
D.sturtevanti	Cornmeal
D.takahashii	Cornmeal
D.teissieri	Cornmeal
D.virilis	Proprionic
H.duncani	Proprionic
S. lativittata	Banana
S.lebanonensis	Proprionic
Z. inermis	Banana
Z. taronus	Banana
Z. tuberculatus	Banana
